# Pandemic Influenza A (H1N1) Virus Infection Increases Apoptosis and HIV-1 Replication in HIV-1 Infected Jurkat Cells

**DOI:** 10.3390/v8020033

**Published:** 2016-02-02

**Authors:** Xue Wang, Jiying Tan, Santanu Biswas, Jiangqin Zhao, Krishnakumar Devadas, Zhiping Ye, Indira Hewlett

**Affiliations:** 1 Lab of Molecular Virology, Division of Emerging and Transfusion Transmitted Diseases, CBER/FDA, Building 72, Rm 4322, 10903 New Hampshire Avenue, Silver Spring, MD 20993, USA; tangjy@lzu.edu.cn (J.T.); Santanu.biswas@fda.hhs.gov (S.B); Jiangqin.zhao@fda.hhs.gov (J.Z.); Krishnakumar.devada@fda.hhs.gov (K.D.); 2 Division of Viral Products, Center for Biologics Evaluation and Research, Food and Drug Administration, Silver Spring, MD 20993, USA; zhiping.ye@fda.hhs.gov

**Keywords:** HIV-1, pandemic influenza A (H1N1) virus, apoptosis, CD4, replication

## Abstract

Influenza virus infection has a significant impact on public health, since it is a major cause of morbidity and mortality. It is not well-known whether influenza virus infection affects cell death and human immunodeficiency virus (HIV)-1 replication in HIV-1-infected patients. Using a lymphoma cell line, Jurkat, we examined the *in vitro* effects of pandemic influenza A (H1N1) virus (pH1N1) infection on cell death and HIV-1 RNA production in infected cells. We found that pH1N1 infection increased apoptotic cell death through Fas and Bax-mediated pathways in HIV-1-infected Jurkat cells. Infection with pH1N1 virus could promote HIV-1 RNA production by activating host transcription factors including nuclear factor kappa-light-chain-enhancer of activated B cells (NF-ĸB), nuclear factor of activated T-cells (NFAT) and activator protein 1 (AP-1) through mitogen-activated protein kinases (MAPK) pathways and T-cell antigen receptor (TCR)-related pathways. The replication of HIV-1 latent infection could be reactivated by pH1N1 infection through TCR and apoptotic pathways. These data indicate that HIV-1 replication can be activated by pH1N1 virus in HIV-1-infected cells resulting in induction of cell death through apoptotic pathways.

## 1. Introduction

It is estimated by the World Health Organization (WHO) that approximately 36.9 (34.3~41.9) million persons worldwide are infected with human immunodeficiency virus (HIV), which is the etiologic agent of Acquired Immunodeficiency Syndrome (AIDS), including 1.7 million in the United States [1]. Influenza A virus can cause acute respiratory infection in humans and animals throughout the world, and has continued to be a significant public health threat, leading to substantial global morbidity and mortality and an average of approximately 23,600 deaths annually in the United States alone [2].

The impact of influenza virus on HIV infection has not been well investigated. Little is known about influenza virus infection in HIV-positive individual. HIV infection has been shown to be related to worse prognosis of influenza. HIV-infected patients in Canada who also had pandemic 2009 influenza A (H1N1) virus (pH1N1) infection had more severe illness than those who did not have the co-infection [3], and fatality was higher than for patients who were not co-infected in California (USA) [4]. Recent reports indicate that adults with AIDS experience substantially elevated influenza-associated mortality [5,6].

Influenza virus infection has been associated with viremia in human and animal models. Viral RNA has been detected in blood in severe human pH1N1 infection [7,8,9]. Encapsidated pH1N1 RNA is stable in blood derived matrices [10] and influenza viruses can be transmitted by blood transfusion in ferrets [11]. Normally, influenza A virus infection is confined to the airways where the virus replicates in respiratory epithelial cells. Cumulated reports indicate that influenza viruses can infect and replicate in blood cells, such as dendritic cells [12], primary monocytes/macrophages, and T cells [13,14].

Infection with HIV-1 can result in apoptotic cell death through activation of both death receptor-mediated and Bax/mitochondrial-mediated apoptotic pathways [15], which cause a progressive depletion of a select group of immune cells namely the CD4+ T helper cells leading to immunodeficiency. While HIV directly and selectively infects CD4+ T cells, the low levels of infected cells in patients is discordant with the rate of CD4+ T cell decline and argues against the role of direct infection in CD4 loss [15]. A viral protein, neuraminidase (NA), derived from the human influenza virus was reported to enhance the level of HIV-1-mediated syncytium formation and HIV-1 replication [16,17]. However, it is not known whether influenza A virus in blood affects HIV-1 replication, or reactivates HIV-1 replication in HIV-1-infected cells. Here, we showed that pandemic influenza A (H1N1) virus infection increased apoptotic cell death and HIV-1 replication in HIV-1 infected Jurkat cells.

## 2. Materials and Methods

### 2.1. Chemicals and Reagents

Rabbit polyclonal antibodies against Bax, Bcl-X_L_ (B-cell lymphoma-extra large), caspase-3, caspase-8, cavoelin-1, CD4, CD28, cytochrome c, FADD (Fas-associated protein with death domain), Fas, FLIP ((FADD-like IL-1β-converting enzyme)-inhibitory protein), p53, Zap-70 (zeta-chain-associated protein kinase 70), and GAPDH (Glyceraldehyde 3-phosphate dehydrogenase were from Santa Cruz Biotechnology (Santa Cruz, CA, USA). AP-1, ERK (extracellular-signal-regulated kinases), JNK (c-Jun N-terminal kinases), p38, NFAT, and NF-kB p65 were bought from Cell Signaling Technology, Inc. (Danvers, MA, USA). All other chemicals were from Sigma (St. Louis, MO, USA).

### 2.2. Cell Culture and HIV-1 Infection

The human Jurkat T cell line (clone JE6.1) and J1.1, a latently HIV infected cell line cloned by limiting dilution from HIV-infected Jurkat cells were obtained from National Institutes of Health (NIH) AIDS reagent program (Germantown, MD, USA) and cultured at 37 °C in 5% CO_2_ in RPMI 1640 medium containing 10% fetal calf serum, 2 mM glutamine, 50 μg/mL penicillin, and 50 μg/mL streptomycin. For HIV-1 infection, Jurkat cells (clone JE6.1) were seeded at 2 × 10^5^ cells/mL for 24 h, and infected with known amounts of HIV-1 (HIV-1 MN strain, 10^9^ copies per 10^6^ cells) for 2 h, washed twice with PBS, and cultured for periods of time indicated.

### 2.3. Pandemic Influenza A (H1N1) Virus Infection

A pandemic influenza A (H1N1) virus (pH1N1) stock, A/California/04/2009, was obtained from the Centers for Disease Control and Prevention (CDC, Atlanta, GA, USA). The virus was propagated in 9–11 day embryonic hen’s eggs. The propagated virus was maintained at −80 °C until use in the study. For virus infection, cells were seeded at 1.5 × 10^6^ cells/well in six-well plates and infected with known amounts (multiplicity of infection, M.O.I) of virus for 2 h, washed three times with PBS, and cultured for periods of time indicated.

### 2.4. Real-Time PCR

Quantitative real-time reverse-transcriptase (RT) PCR was used for quantitation of viral RNA. Viral RNA was isolated from 140 µL of culture supernatant by using the QIAamp Viral RNA Mini Kit (Qiagen Inc., Valencia, CA, USA) according to the manufacturer’s protocol. The primers and TaqMan probe were designed in the *gag* capsid (p24) region, which is the variable region among most of the HIV-1 subtype B isolate sequences according to GenBank database. The forward primer was 5′-GACATCAAGCAGCCATGCAA-3′, corresponding to nucleotides 1367–1386, and the reverse primer was 5′-CTATCCCATTCTGCAGCTTCCT-3′, corresponding to nucleotides 1430–1409. The TaqMan probe was oligonucleotide 5′-ATTGATGGT CTCTTTTAACA-3′, corresponding to nucleotides 1488–1507, coupled with a reporter dye (6-carboxy fluorescein) (FAM) at the 5′ end and a non-fluorescent quencher and a minor groove binder (MGB), which is a Tm enhancer, at the 3′ end. The nucleic acids were amplified and detected in an automated TaqMan 7500 Analyzer by using QuantiTect™ Probe RT-PCR kit (Qiagen Inc.). The 25-µL PCR mixture consisted of 100 nM primers and 100 nM probe. Following three thermal steps at 55 °C for 5 min, at 50 °C for 30 min, and at 95 °C for 10 min. 45 cycles of two-step PCR at 95 °C for 15 s and at 60 °C for 1 min were performed.

The data are expressed as copy numbers/mL. Known concentrations of HIV-1 (MN) viral RNA (serially diluted: 10^8^ to 100 copies) were used as templates and quantitative RT-PCR performed to generate a standard curve. Each value represents the average concentration of six reactions in triple isolated repeats based on the standard curve.

### 2.5. Caspase-3 Assay

EnzChek^®^ Caspase-3 Assay Kit #1, Z-DEVD-AMC Substrate from Invitrogen (Grand Island, NY, USA) was used to test caspase-3 activity, which was performed according to the manufacturer’s instruction.

### 2.6. Cell Viability Assay

Cell viability was determined by Trypan blue exclusion analysis (Life Technologies, Waltham, MA, USA).

### 2.7. Subcellular Protein Fractionation

To generate membrane and cytoplasmic lysates, Subcellular Protein Fractionation Kit for Cultured Cells was used (Thermo Scientific, Rockford, IL, USA). Lysis of cells generated membrane and cytoplasmic protein extracts. The protocol was performed according to the manufacturer’s instructions. Normalized portions of each extract (10 µg) were analyzed by Western blotting using specific antibodies against proteins from various cellular compartments, including cytoplasmic (cytochrome c) and plasma membrane (caveolin-1) (Figure S1).

### 2.8. Western Blot Analysis

Proteins were isolated from the culture of Jurkat cells with RIPA buffer (1 × PBS, 1% (*v*/*v*) NP-40, 0.5% (*w*/*v*) sodium deoxycholate, 0.1% (*w*/*v*) SDS, 0.1 mg/mL PMSF, 30 μL/mL aprotinin, 1 mM sodium orthovanadate). Equal amounts of protein were boiled in the loading buffer (100 mM Tris-HCl, 200 mM DTT, 4% SDS, 0.2% bromphenol blue, 20% glycerol) and separated on SDS-PAGE and blotted onto polyvinylidene difluoride membranes. For immunoprecipitation (IP), 1 µg of antibody was added to 500 µg of total protein in 500 µL, rotated for 2 h at 4 °C, then incubated with 20 µL of protein A-sucrose beads (Santa Cruz Biotechnology) for another 2 h, spun down at 500 × *g*, and washed three times with RIPA buffer. The data represented are from three independent experiments.

### 2.9. Statistical Analysis

The unpaired Student’s *t* test was used for data analyses as indicated, and a value of *p* < 0.01 was considered very significant (**).

## 3. Results

### 3.1. Pandemic 2009 Influenza A (H1N1) Virus (pH1N1) Infection Induction of Apoptotic Death in HIV-1 Infected Cells

**Figure 1:**
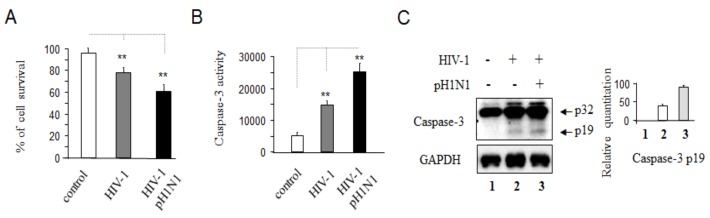
Pandemic influenza A (H1N1) virus infection can induce cell death through apoptosis in HIV-1-infected cells. Jurkat cells were infected with HIV-1 (MN) for 3 days and then infected with a pandemic influenza A (H1N1) virus (pH1N1) stock, A/California/04/2009, and cultured for another 3 days. Cells incubated without viruses were used as control. (**A**) Cell viability relative to control was determined by Trypan blue exclusion; (**B**) The total cell lysates were subjected to test caspase-3 activities with EnzChek® Caspase-3 Assay Kit #1-Z-DEVD-AMC Substrate from Invitrogen (grand Island, NY, USA), which was performed according to the manufacturer’s instruction; (**C**) The total cell lysates with RIPA buffer were subjected to Western blot analysis to detect caspase-3.The unpaired Student’s *t* test was used for data analyses and a value of *p* < 0.01 was considered very significant (**) relative to control.

It was reported that Influenza virus infection could cause severe complications in HIV-1-infected individuals leading to an increased risk of complications and death compared with uninfected individuals [5,6]. We found that pH1N1 could infect and replicate in Jurkat cells and primary CD4+ T cells (Figure S2). To examine whether pH1N1 infection increased cell death due to HIV-1 infection, Jurkat cells were infected with HIV-1 for three days, followed by incubation in the presence of pH1N1 for another three days. As shown in Figure 1A, pH1N1 infection caused increased cell death in HIV-1 infected cells compared with uninfected cells (control). We also tested caspase-3 activities (Figure 1B,C) and found that more caspase-3 was activated by pH1N1 in HIV-1 infected cells than uninfected cells (control). We also obtained the similar results with using primary CD4 T cells incubated with HIV-1 for seven days and then infected with pH1N1 for another three days (Figure S3A,B).

These data indicated that pH1N1 infection caused cell death through apoptotic signaling pathways in T cells.

### 3.2. Pandemic Influenza A (H1N1) Virus (pH1N1) Infection Could Activate Both Apoptotic Pathways

**Figure 2:**
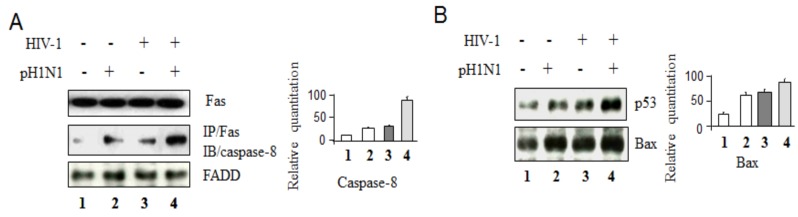
Pandemic influenza A (H1N1) virus infection can induce more intense activation of apoptotic pathways in HIV-1 infected cells. Jurkat cells were infected with/without HIV-1 (MN) for 3 days, and then infected with pH1N1, stock A/California/04/2009, and cultured for 3 days. The cell lysates were subjected to Western blot analysis to detect FADD (**A**), p53 and Bax (**B**), with Fas (A) and GAPDH (B) as the standard loading control. DISC formation was evaluated by immunoprecipitation (IP) total lysates with anti-Fas antibody followed by immunoblotting (IB) to detect caspase-8 (A).

Recently, we found that pH1N1 is able to induce apoptotic cell death in A549 cells [18]. To determine whether pH1N1 can promote death through Fas-mediated apoptotic pathway in Jurkat cells, total cell lysates from cells infected with HIV-1 followed by infection with pH1N1 for an additional three days were used for IP with anti-Fas antibody and Western blotting with anti-caspase-8 antibody to evaluate death-inducing signaling complex (DISC) formation. As shown in Figure 2A, more DISC formation was found with the pH1N1/HIV-1 infection relative to HIV-1 infection or pH1N1 alone. Figure 2B showed that high level of expression of p53 and Bax proteins was detected in Jurkat cells infected with pH1N1 infection and HIV-1-infected cells, compared with pH1N1 or HIV-1 alone.

These data indicate that pandemic influenza A (H1N1) infection can induce both Fas-mediated and Bax-mediated apoptotic pathways. A higher level of activation of both apoptotic signaling pathways was found in pH1N1 infected Jurkat cells after HIV-1 infection.

### 3.3. Pandemic Influenza A (H1N1) Infection Enhanced HIV-1 Replication

**Figure 3:**
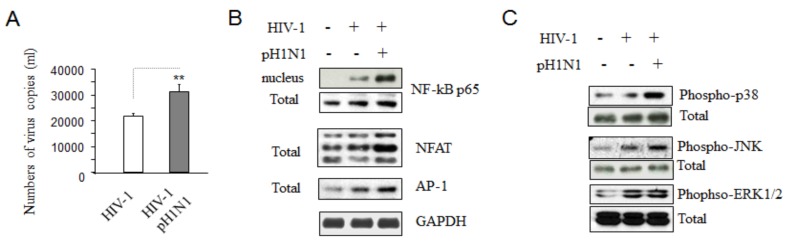
Pandemic influenza A (H1N1) virus infection can activate more HIV RNA production through increased activities of some host transcript factors in HIV-1 infected cells. Jurkat cells were infected with HIV-1 (MN) for 3 days, and then infected with pH1N1, stock A/California/04/2009, and cultured for 3 days. (**A**) 140 μL of culture supernatants containing HIV-1 particules were used to isolate viral RNA. 10 μL in 50 μL of the RNA was used as template to perform real-time PCR. Known concentrations if HIV-1 (MN) viral RNA (serially diluted: 10^8^ to 100 copies) were used as template and quantitative RT-PCR performed to generate a standard curve. Each value represents the average concentration of six reactions in triple isolated repeats based on the standard curve. The cell lysates were subjected to Western blot analysis to detect NF-κB p65, NFAT, AP-1 (**B**) and p38, JNK, ERK1/2 (**C**) with GAPDH as the standard.

Recent reports indicate that pH1N1 infection was associated with increase in mortality in patients with late and advanced HIV disease [5,6] and increased cell death was found in pH1N1-infected Jurkat cells after HIV-1 infection (Figure 1A). We determined whether pH1N1 infection could influence HIV-1 replication. As shown in Figure 3A, Jurkat cells infected with HIV-1 and incubated with pH1N1 for another three days, had higher levels of HIV-1 RNA relative to cell with HIV-1 infection alone, suggesting that pH1N1 infection increases HIV-1 replication in co-infected cells in Jurkat cells and in CD4+ T cells (Figure S3C).

It has been well-known that some host transcription factors are required for HIV replication, including nuclear factor kappa-light-chain-enhancer of activated B cells (NF-κB), NFAT, AP-1, which are up-regulated when T-cells become activated [19,20,21]. They stimulate viral gene expression through transcription with the long terminal repeat (LTR) of HIV-1 [22]. Cells treated with pH1N1 had higher level of NF-ĸB phosphorylation and increased protein expression of NFAT and AP-1 (Figure 3B) relative to HIV-1 infection alone, suggesting pH1N1 infection can activate host transcription factors required for HIV-1 replication in Jurkat cells.

The mitogen-activated protein kinases (MAPKs) are central components of signal transduction pathways activated by diverse extracellular stimuli. Several studies have shown that the MAPK signal pathway can positively regulate replication of HIV-1, and pathway inhibitors can block HIV-1 replication [23,24]. Our results indicate that pH1N1 infection promotes MAPK pathway molecules, such as p38, ERK, and JNK phosphorylation (Figure 3C).

These data approve the conclusion that pandemic influenza A (H1N1) infection increases HIV-1 replication in infected cells through activation of NF-ĸB, NFAT, AP-1, and MAPK pathways that are necessary for HIV-1 replication.

### 3.4. Pandemic Influenza A (H1N1) Infection Activated TCR Signaling in HIV-1 Infected Cells

**Figure 4:**
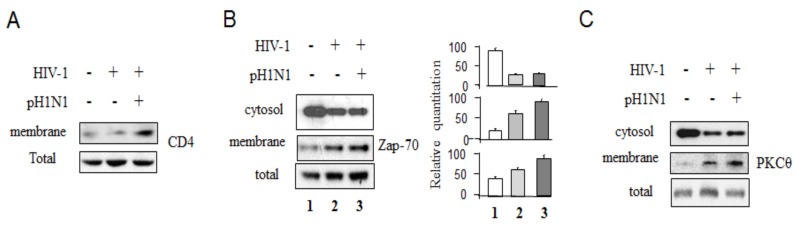
Pandemic influenza A (H1N1) virus infection can induce more CD4 epresiion and activate TCR signaling pathways in HIV-1-infected cells. Jurkat cells were infected with HIV-1 (MN) for 3 days, and then infected with pH1N1, stock A/California/04/2009, and cultured for 3 days. The cells lysates were used to subcellular protein fractionation and Western blot analysis to detect CD4 in plasma membrane (**A**); Zap-70 in cytosol amd plasma membrane (**B**); and PKCɵ in cytosol and membrane (**C**), with their total lysates as standards.

It is well known that CD4 is the receptor of HIV-1 [25,26]. HIV-1 gp120 binds to the CD4 molecule on the surface of host cells [24]. Since HIV-1 entry takes place in lipid rafts of the plasma membrane [27], we examined whether pH1N1 infection affects CD4 expression on the plasma membrane in Jurkat cells infected with HIV-1 and pH1N1. As shown in Figure 4A, pH1N1 infection increased CD4 expression in the plasma membrane of HIV-1-infected Jurkat cells compared with HIV-1 infection alone.

The T cell activation and proliferation control HIV-1 replication and gene expression [28]; and activation of T cells through involvement of TCR, CD3/CD8, or CD28, can trigger and activate downstream molecules, such as Zap-70 and PKCɵ, and which activate transcription factors, AP-1 NFAT and NF-κB [19,29], and increase HIV-1 gene expression and replication directed by the HIV-1 LTR [22]. Activation events of T cells require association with lipid rafts of the plasma membrane [24,27]. Our results showed that pH1N1 infection increased more Zap-70 and PKCɵ expression on plasma membranes relative to HIV-1 infection alone (Figure 4B,C).

These data indicate that pandemic influenza A (H1N1) infection can increase accumulation of CD4 protein and induce T cell signaling and activate host transcription factors required for HIV-1 replication.

### 3.5. Pandemic Influenza A (H1N1) Infection Reactivated HIV-1 Replication in Latently Infected Cells

**Figure 5:**
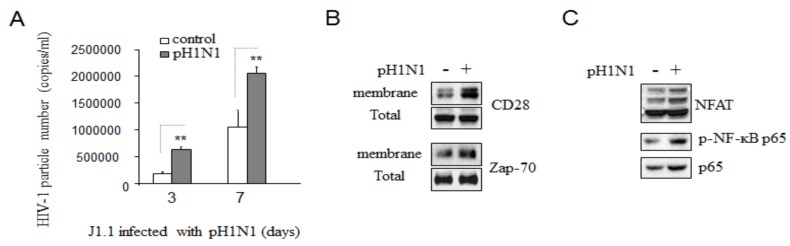
Pandemic influenza A (H1N1) virus infection can reactivate HIV-1 replication from its latent infections. J1.1 cells were infected with pH1N1, stock A/California/04/2009, and cultured for 3 days. (**A**) 140 μL of culture supernatants containing HIV-1 particles were used to isolate viral RNA. 10 μL in 50 μL of the RNA was used as template to perform real-time PCR. Known concentration of HIV-1 (MN) viral RNA (serially diluted: 10^8^ to 100 copies) were used as templates and quantitative RT-PCR performed to generate a standard curve. Each value represents the average concentration of six reactions in triple isolated repeats based on the standard curve. (**B**) The cell lysates were used to subcellular protein fractionation and Western blot analysis to detect CD28 and Zap-70 in plasma membrane, with their total lysates as standards. (**C**) The total cell lysates were used to Western blot analysis to detect NFAT and NF-κB p65.

HIV-1 infection is presently readily controlled with combination antiretroviral therapy. The persistence of HIV-1 in the face of potent antiretroviral drugs appears to be largely due to the ability of the virus to establish a state of latent infection in a long-lived population of resting memory CD4+ T cells [30]. This latent reservoir is widely considered to be the major barrier to curing infection and is currently the subject of intense research [30]. To determine whether pH1N1 infection can reactivate HIV-1 latent infection, J1.1 cells were infected with pH1N1 for three or seven days days. As shown in Figure 5A, measure with real-time PCR analysis showed that significant high level of HIV-1 RNA production was detected in pH1N1 infected cell relative to non-pH1N1 infected controls.

Cell fractionation showed that pH1N1 infection caused increased accumulation of CD28 and Zap-70 proteins in plasma membrane in J1.1 cells (Figure 5B). Western blotting analysis showed that pH1N1 infection increased NFAT expression and activated NF-ĸB signaling (Figure 5C).

**Figure 6:**
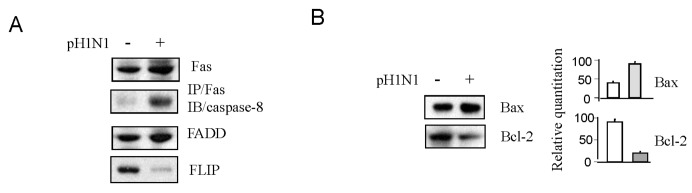
Pandemic influenza A (H1N1) virus infection can activate apoptotic pathways in HIV-1 latent infected cells. J1.1 cells were infected with pH1N1, stock A/California/04/2009, and cultured for 3 days. (**A**) The cell lysates were subjected to Western blot analysis to detect FADD, FAS and FLIP (A), Bax and BCL-2 (**B**). DISC formation was evaluated by IP total lysates with anti-Fas antibody followed by IB to detect caspase-8 (A).

pH1N1 infection could activate both apoptotic pathways with an increased expression of pre-apoptotic proteins (such as Fas, DISC formation, FADD, and Bax) and a decreased expression of anti-apoptotic proteins (such as FLIP and Bcl-2) (Figure 6), suggesting that apoptotic pathways may be also involved in pH1N1-induced reactivation of HIV-1 replication. These data indicate that pandemic influenza A (H1N1) infection is able to reactivate latent HIV-1 infection *in vitro*.

## 4. Discussion

During the 2009 H1N1 influenza A virus pandemic, it was reported that some patients developed severe pneumonia leading to acute respiratory distress syndrome (ARDS) and multiple organ dysfunction, associated with high (17%–54%) mortality [31,32]. However, there is little information available on the direct interaction between influenza virus and the host immune system that pertains to severe disease outcomes. It has been reported that pathogenesis due to influenza infections was associated with alterations in the lymphohematopoietic system, leading to the destruction of lymphocytes and histopathological necrosis of lymphoid tissues [33,34]. There is little information available on whether and how influenza infection could damage the immune system directly and alter its ability to control other infectious pathogens that ultimately are detrimental to the host. Our study showed that pandemic influenza A (H1N1) virus infection is able to enhance cell death in HIV-1-infected cells (Figure 1).

HIV-1 infection may be associated with a worse prognosis of influenza and adults with AIDS experience substantially elevated influenza-associated mortality, which has declined with widespread introduction of HAART (highly-active anti-retroviral therapy), but not completely eliminated [35]. In HIV patients, well controlled on HAART, pandemic influenza A (H1N1) virus infection has resulted in a similar clinical outcome and prognosis to that of non-HIV patients [34] and oseltamivir treatment has been known to shorten the duration of influenza viral shedding in children [36] and adults [37,38]. CD4 cell count and plasma HIV-1 RNA did not differ before and 4–6 weeks after influenza A H1N1 diagnosis in the presence of oseltamivir treatment in the HIV-positive group [37]. However, HIV-1-infected subjects that had progressed to immune deficiency presented an increased mortality by pH1N1 [5]. This information suggests that HIV-1 activation, induced by influenza virus infection, can be controlled by oseltamivir in “normal” HIV-positive persons on HAART treatment, and may not be controlled in HIV-infected patients under illness condition [5].

Influenza A virus induces apoptotic death in infected epithelial, lymphocyte, and phagocytic cells [18,33,34,39], and apoptosis is a source of tissue damage during infection [33]. Distal lung epithelial cell apoptosis and apoptosis-resulting in necrosis were described as a classical feature of H5N1- or pandemic H1N1-induced ARDS [40]. Epithelial cell apoptosis is generally seen as a major underlying cause of diffuse alveolar damage in many forms of ARDS [40]. So far, it has been clear that influenza infection can affect both intrinsic and extrinsic apoptotic pathways [18]. It was also reported that after influenza virus infection, some CD4+ cells in the spleen and thymus may express viral proteins [41], and a population of T cells is susceptible to influenza A virus in infected mice [42,43]. Influenza virus infection has been shown to activate TCR-signaling pathways in Jurkat cells [16] and pandemic influenza A (H1N1) virus infection causes apoptotic death in HIV-1-infected Jurkat cells (Figure 4 and Figure 5B,C). Previously, we reported that pre-apoptotic molecules from apoptotic pathways increase HIV-1 replication, while anti-apoptotic proteins inhibit viral RNA yield [23]. Consistent with our previous observation, pandemic influenza A (H1N1) virus infection can increase HIV-1 replication with up-regulation of pro-apoptotic molecules, down-regulation of anti-apoptotic proteins, and with an increased activation of T cells through TCR-related signaling pathways (Figure 1C, Figure 2 and Figure 6) required for T- cell activation and HIV-1 replication; however the detailed mechanisms by which pandemic influenza A (H1N1) virus infection induces HIV-1 replication needs further study.

In conclusion, our results demonstrate that pandemic influenza A (H1N1) virus infection can induce cell death through apoptotic signaling pathways and promote HIV-1 replication through the MAPK and TCR-related signaling pathways in HIV-1-infected Jurkat cells. Pandemic influenza A (H1N1) virus infection is also able to reactivate HIV-1 replication from its state of latent infection through activating apoptosis and TCR-signaling pathways.

## Supplementary Files

Supplementary File 1
