# Supplementary material for: Pandemic Influenza A (H1N1) Virus Infection Increases Apoptosis and HIV-1 Replication in HIV-1 Infected Jurkat Cells
Source: Viruses. 2016 Feb 2;8(2):33. doi: 10.3390/v8020033 (PMC4776188; doi:10.3390/v8020033)
Supplement: Supplementary File 1 [file viruses-08-00033-s001.pdf]

Supplementary Materials: Pandemic Influenza A (H1N1) Virus Infection Increases Apoptosis and HIV-1 Replication in HIV-1 Infected Jurkat Cells

Xue Wang [1,†,*], Jiying Tan [1,3,†], Santanu Biswas [1], Jiangqin Zhao [1], Krishnakumar Devadas [1], Zhiping Ye [2] and Indira Hewlett [1,*]

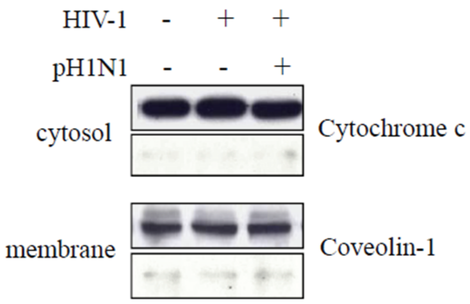

Figure S1. Subcellular protein fractionation. To generate membrane and cytoplasmic lysates, Subcellular Protein Fractionation Kit for Cultured Cells was used (Thermo Scientific, Rockford, IL, USA). Lysis of cells generated membrane and cytoplasmic protein extracts. The protocol was performed according to the manufacturer's instructions. Normalized portions of each extract (10 μg) were analyzed by Western blotting using specific antibodies against proteins from various cellular compartments including cytoplasmic (cytochrome c) and plasma membrane (caveolin-1) as indicated in Figure.

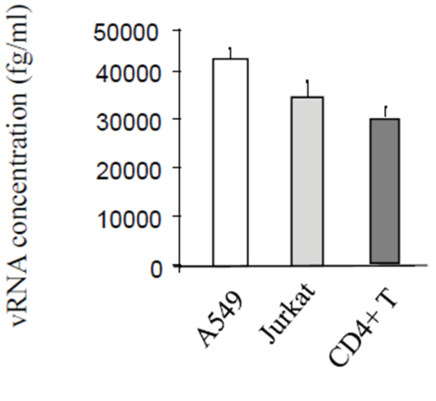

Figure S2. Pandemic influenza A (H1N1) virus can infect and replicate in Jurkat cells and primary CD4 T cells. A549 cells (as control), Jurkat cells, or human primary CD+ T cells (Astarte Biologics, LLC, Bothell, WA, USA) were infected with 1 MOI of pandemic influenza A (H1N1) virus, A/California/04/2009, for 2 hours, washed three time with PBS, and then cultured for three days. 140 μL of the culture supernatants containing influenza viral particles were used to isolate viral RNA. 5 μL in 50 μL of the RNA was used as template to perform real-time PCR. The PCR were performed with a set of primers and probes for the matrix gene, M, of the H1N1 influenza A virus, according to the GenBank database. The forward primer was 5' CGTCAGGCCCCCTCAAA 3', the reverse primer was 5' TTTCCTGCAAAGAC-ACTTTCCA 3', and the TaqMan probe was oligonucleotide 5' CGAGATCGCGCA-GAGA 3'. The data are displayed here as viral concentration, Femtogram (fg) per mL. Known amounts of A/California/04/2009 viral RNA (serially diluted: 10^8 to 10 fg) were used as templates and quantitative RT-PCR performed to generate a standard curve. Each value represents the average concentration of six reactions in triple isolated repeats based on the standard curve. It showed that pandemic influenza A (H1N1) virus could infect and replicate Jurhat cells or human primary CD4 T cells.

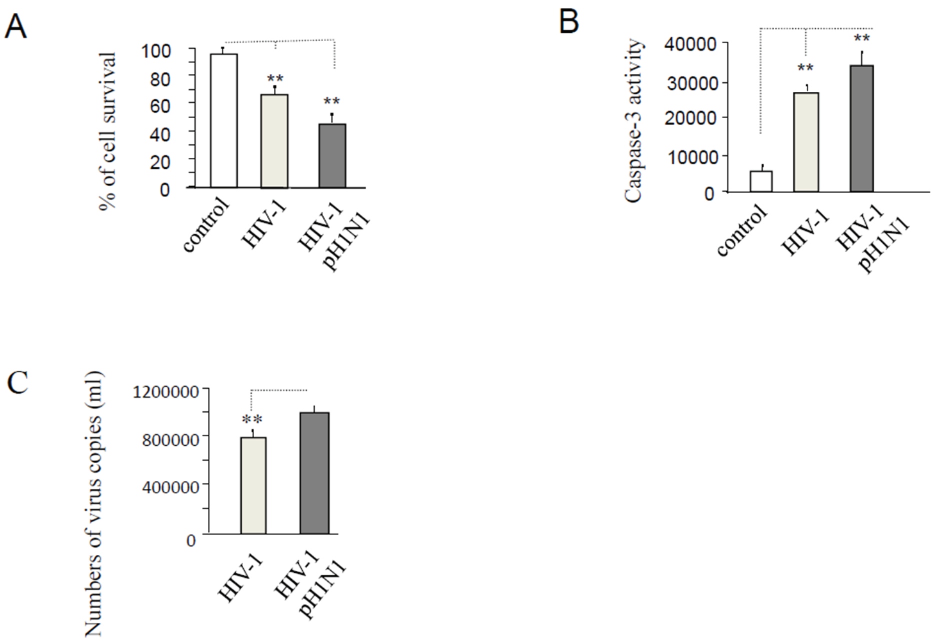

Figure S3. Pandemic influenza A (H1N1) virus infection can induce cell death through apoptosis and activate more HIV-1 RNA production in HIV-1-infected CD4+ T cells. Primary CD+ T cells (Astarte Biologics, LLC, Bothell, WA) were infected with HIV-1 (MN) for seven days, and then infected with a pandemic influenza A (H1N1) virus (pH1N1) stock, A/California/04/2009, and cultured for three days. (**A**) Cell viability relative to control was determined by trypan blue exclusion; (**B**) The total cell lysates were subjected to test caspase-3 activities with EnzChek® Caspase-3 Assay Kit #1-Z-DEVD-AMC Substrate from Invitrogen (Grand Island, NY, USA), which was performed according to the manufacturer's instruction; (**C**) 140 μL of culture supernatants containing HIV-1 particles were used to isolate viral RNA. 10 μL in 50 μL of the RNA was used as template to perform real-time PCR. Known concentrations of HIV-1 (MN) viral RNA (serially diluted: 10^8 to 100 copies) were used as templates and quantitative RT-PCR performed to generate a standard curve. Each value represents the average concentration of six reactions in triple isolated repeats based on the standard curve. The unpaired Student's *t* test was used for data analyses and a value of $p < 0.01$ was considered very significant (**) relative to control. We found that pandemic influenza A (H1N1) virus infection increases apoptosis (cell survival curve and caspase-3 activities) and HIV-1 replication (HIV-1 RNA production) in HIV-1-infected primary CD4 T cells.

© 2016 by the authors; licensee MDPI, Basel, Switzerland. This article is an open access article distributed under the terms and conditions of the Creative Commons by Attribution (CC-BY) license (http://creativecommons.org/licenses/by/4.0/).
